# Accuracy of a subcutaneous continuous glucose monitor compared with an intravenous continuous glucose monitor in an intensive care unit

**DOI:** 10.1186/2197-425X-3-S1-A292

**Published:** 2015-10-01

**Authors:** Y Takahashi, T Yatabe, M Munekage, M Sakaguchi, A Nishigaki, M Yokoyama, K Hanazaki

**Affiliations:** Department of Anesthesiology, Kochi Medical School, Nankoku, Japan; Department of Surgery, Kochi Medical School, Nankoku, Japan

## Introduction

Blood glucose management is important in the intensive care unit (ICU). Although we have used an intravenous continuous glucose monitor for blood glucose management to avoid hypoglycemia, there has been a problem with interruption of blood glucose measurement caused by blood removal failure [1]. A previous study reported that a subcutaneous continuous glucose monitor was reliable for use in critically ill patients [2].

## Objectives

We thought that a subcutaneous continuous glucose monitor was more useful compared with an intravenous continuous glucose monitor because blood removal failure may not occur. However, given the lack of previous comparative studies, the purpose of this study was to compare the subcutaneous and intravenous continuous glucose monitors.

## Methods

This was an observational trial, registered in the University Hospital Medical Information Network Clinical Trial Registry System (UMIN-CTR, ID:000013338). The ethics committee of our hospital approved the study, and patient informed consent was obtained. We included patients who were admitted to our ICU after hepato-biliary pancreatic surgery. A 20-G intravenous catheter was inserted into a peripheral vein and connected to the intravenous continuous glucose monitor STG-55 (Nikkiso, Tokyo, Japan). The subcutaneous continuous glucose monitor iPr02 (Medtronic Japan, Tokyo, Japan) was inserted into the subcutaneous tissue of the ipsilateral upper arm. Continuous blood glucose measurement was performed between ICU admission and discharge. The STG-55 measured the glucose level in real time, and the iPr02 measured this every 5 minutes. We compared glucose levels obtained using the two devices every 5 minutes using a Bland-Altman plot and a regression analyses.

## Results

A total of 19 patients were enrolled. Of these cases, the STG-55 broke down in 3 cases, and the iPr02 did not export data in one case; thus, 15 cases were analyzed. We collected 3145 comparative samples. Of these samples, 158 were excluded because the STG-55 could not measure blood glucose owing to blood removal failure (4.5% of all samples). The mean glucose level measured using the STG-55 was 142 ± 18 mg/dl, and that measured using the iPr02 was 145 ± 31 mg/dl. A linear regression line had the equation of the form y = 0.407x+87.556. The coefficient of determination was 0.05 and p < 0.05 by the F-test. The mean of the differences was −3.6 mg/dl, with a 95% agreement limit of −68 to +61 mg/dl. The percent error was 46%.

## Conclusions

Our study suggests that the accuracy of the subcutaneous continuous glucose monitor was lower than that of the intravenous continuous glucose monitor in ICU patients. However, we considered that both devices need improvement because blood removal failure was observed in 4.5% of all samples with the intravenous continuous glucose monitor.
